# *Clitocybe nuda* Activates Dendritic Cells and Acts as a DNA Vaccine Adjuvant

**DOI:** 10.1155/2013/761454

**Published:** 2013-08-22

**Authors:** Mei-Hsing Chen, Wei-Sung Li, Yun-Sheng Lue, Ching-Liang Chu, I-Hong Pan, Ching-Huai Ko, Der-Yuan Chen, Ching-Hsiung Lin, Sheng-Hao Lin, Chih-Peng Chang, Chi-Chen Lin

**Affiliations:** ^1^Plant Pathology Division, Taiwan Agricultural Research Institute (TARI), Council of Agriculture (COA), Executive Yuan, Wufeng 413, Taiwan; ^2^Graduate Institute of Immunology, National Taiwan University, Taipei 112, Taiwan; ^3^Biomedical Technology and Device Research Laboratories, Industrial Technology Research Institute, Hsinchu 300, Taiwan; ^4^Institute of Biomedical Science, National Chung-Hsing University, Taichung 402, Taiwan; ^5^Faculty of Medicine, National Yang-Ming University, Taipei 112, Taiwan; ^6^Division of Allergy, Immunology and Rheumatology, Taichung Veterans General Hospital, Taichung 407, Taiwan; ^7^Division of Chest Medicine, Department of Internal Medicine, Changhua Christian Hospital, Changhua 500, Taiwan; ^8^Department of Microbiology and Immunology, College of Medicine, National Cheng Kung University, Tainan 701, Taiwan; ^9^Department of Medical Research and Education, Taichung Veterans General Hospital, Taichung 407, Taiwan

## Abstract

This work represents the first evaluation of the effects of water extract of *C. nuda* (WE-CN), an edible mushroom, on murine bone marrow-derived dendritic cells (BMDCs) and the potential pathway through which the effects are mediated. Our experimental results show that WE-CN could induce phenotypic maturation of DCs, as shown by the increased expression of MHC and costimulatory molecules. In addition, it also induced the proinflammatory cytokines expression on DCs and enhanced both the proliferation and IFN-**γ** secretion of allogenic T cells. Therefore, since WE-CN did not induce maturation of DCs generated from mice with mutated TLR-4 or TLR-2, suggesting that TLR4 and TLR2 might function as membrane receptors for WE-CN. Moreover, the mechanism of action of WE-CN may be mediated by increased phosphorylation of ERK, p38, and JNK mitogen-activated protein kinase (MAPK) and increased NF-**κ**B p65 activity, which are important signaling molecules downstream of TLR-4 and TLR-2. Finally, coimmunization of mice with WE-CN and a HER-2/neu DNA vaccine induced a HER-2/neu-specific Th1 response that resulted in significant inhibition of HER-2/neu overexpressing mouse bladder tumor (MBT-2) growth. These data suggest that WE-CN induces DC maturation through TLR-4 and/or TLR-2 and that WE-CN can be used as an adjuvant in cancer vaccine immunotherapy.

## 1. Introduction 

Dendritic cells (DCs) are professional antigen-presenting cells (APCs) that play a key role as immune sentinels by initiating T-cell responses and linking innate and adaptive immunity [1, 2]. DCs are present at different stages of maturation in the circulation and in nonlymphoid and lymphoid organs. DCs reside in an immature form in peripheral nonlymphoid tissues, where they capture and process exogenous antigens [3]. Once activated, DCs migrate to the T-cell-dependent areas of secondary lymphoid organs, where they present antigenic peptides to T lymphocytes and stimulate naïve T-cell responses through cytokine signals, major histocompatibility complex- (MHC-) presenting Ag peptides and costimulatory molecules (e.g., CD40 and CD80). As key regulatory mediators of immune responses, DCs are being pursued for the development of potent new vaccines against cancer and infectious diseases [4, 5]. In addition, the identification of materials that can modulate DC activation and function is an emerging field that can develop alongside DC immunobiology [6]. Natural or synthetic activators that promote DC activation may potentially be candidate adjuvants for application in immunotherapy and vaccination.

Growing evidence-based research has suggested benefits of consuming mushrooms as a functional food or through the use of extracted bioactive compounds as dietary supplements, immunomodulators (biological response modifiers), and adjuvant tumor therapy [7–10]. Numerous compounds have been isolated from mushrooms and have great potential for development as mushroom nutraceutical and pharmaceutical products. Among these compounds, water-soluble polysaccharides and proteoglycans, proteins, and various constituents of small molecular mass are considered to have immunomodulatory potential by regulating several types of immune cells that function in antitumor or antimicrobial activities, including dendritic cells, macrophages, cytolytic T cells, and NK cells [9–12].

*Clitocybe nuda* (also known as *Lepista nuda*, commonly known as blewits) is an edible woodland mushroom found in Europe, North America, Asia, and Australia [13]. Due to its special fragrance and delicate texture, *C. nuda* has been cultivated in France, Holland, Britain, and Taiwan. Several bioactive extracts from *C. nuda* have been found to exhibit antioxidant and antimicrobial properties [14–18], but few reports have described medicinal activities or health benefit in human disorders. To our knowledge, only three papers have shown that *C. nuda* extract affects cancer cells in vitro [19–21]. However, no studies have specifically reported immunologic effects of *C. nuda*.

Therefore, to study the effects of *C. nuda* on immune response and its potential cellular targets, we investigated whether *C. nuda* affects the maturation and functional properties of murine bone marrow-derived dendritic cells (BMDCs). Our findings demonstrate for the first time that water extract *Clitocybe nuda* (WE-CN) induces the phenotypic and functional maturation of BMDCs via ERK1/2, JNK, and p38 MAPK and the nuclear translocation of the NF-*κ*B p65 subunit, in part, by the TLR-4 and TLR-2 pathways. Significantly, WE-CN cotreatment enhanced the therapeutic efficacy of a HER-2/neu DNA vaccine against HER-2/neu-overexpressing tumors, suggesting that WE-CN could be a novel adjuvant and has potential application in cancer therapy and vaccination.

## 2. Material and Methods

### 2.1. Mice and Cell Cultures

Five- to eight-week-old specific pathogen-free female C57BL/6, C3H/HeN, and C3H/HeJ (TLR-4 mutant) mice were purchased from the National Laboratory Animal Center (Taipei, Taiwan). TLR-2 knockout mice were provided by Dr. Chih-Peng Chang (NCKU, Tainan, Taiwan). OT-I TCR transgenic mice were purchased from the Jackson Laboratory (Bar Harbor, ME, USA). OT-II TCR transgenic mice were provided by Dr. Clifford Lowell (UCSF, San Francisco, CA, USA). All mice were housed in the barrier facility at Taichung Veterans General Hospital (Taichung, Taiwan) in accordance with the Institutional Animal Care and Use Committee guidelines for animal experimentation, and all procedures were performed in accordance with the Institutional Animal Care and Use Committee guidelines for animal experimentation. 

The MBT-2 murine bladder tumor cell line, derived from a carcinogen-induced bladder tumor in C3H mice, has been described and is known to express high levels of p185^neu^ [22]. Mouse DCs were generated from bone marrow as previously described [23]. Bone marrow (BM) cells were flushed from the femurs and tibias of C57BL/6 mice, lyzed red blood cells with ammonium chloride, and then washed with PBS. BM cells were suspended in RPMI-1640 medium supplemented with 10% heat-inactivated fetal bovine serum, 100 U/mL penicillin G, 100 *μ*g/mL streptomycin, 2 mM L-glutamine, 20 ng/mL recombinant mouse granulocyte-monocyte colony-stimulating factor (Peprotech) and 20 ng/mL recombinant mouse IL-4 (Peprotech), and culturedin 24-well plates (5 × 10^5^ cells/mL). Fresh medium was supplied every 2 days, and nonadherent cells were harvested on day 7 as immature DCs.

### 2.2. Preparation of Water Extract *C. nuda*

The fruiting bodies of *C. nuda* strain Tainung No. 1 were cultivated on compost and harvested by the Taiwan Agricultural Research Institute Mushroom Laboratory. After oven-drying, 30 g of the dried mushroom samples were milled and submitted to aqueous extraction under reflux (40x at 100°C for 40 min). The aqueous extract was filtered over Whatman no. 1 paper, and the filtrate was evaporated to a small volume and lyophilized. The dry extracts were stored frozen at −20°C until use. The crude extracts were resolubilized in MilliQ water at 4 different concentrations (12.5, 25, 50, and 100 *μ*g/mL). To remove endotoxins (lipopolysaccharides or LPS), the sample preparations were passed through an EndoTrap Blue column (Hyglos, Bernried, Germany). The level of endotoxin in the sample preparation was determined by a quantitative, chromogenic QCL-1000 Limulus amoebocyte lysate (LAL) assay (Cambrex Bio Science Walkersville, Inc., Walkersville, MD, USA) and was found to be <0.1 ng/mg protein. Furthermore, to inhibit endotoxin activity, the samples were incubated on a rotator for 2 hours at 37°C with 10 *μ*g/mL polymyxin B.

### 2.3. In Vitro DC Activation

Immature DCs were cultured at a density of 1 × 10^6^ cells/well in 24-well plates in medium alone or in the presence of 100 ng/mL LPS (*Escherichia coli*, serotype O26:B6 (Sigma St. Louis, MO, USA)), 1 *μ*g/mL lipoteichoic acid (LTA, LTA; L2515, from Staphylococcus aureus, SIGMA, St. Louis, MO, USA), or WE-CN (0, 25, 50, or 100 *μ*g/mL) for 24 hr (or 6 hr for the TNF-alpha ELISA). The cells were then used for further immunophenotyping, the cytokine production assay, or the addition of T cells for DC-T coculture experiments.

### 2.4. Flow Cytometric Analysis of Surface Markers

After stimulation, DCs were harvested and stained with FITC-conjugated mouse CD11c^+^ or phycoerythrin- (PE-) conjugated anti-mouse CD40, anti-mouse CD80, anti-mouse CD86, anti-mouse MHC class I, anti-mouse MHC class II, or isotype-matched control mAbs (all from BioLegend, San Diego, CA, USA) for 45 min on ice (1 *μ*g/mL diluted in PBS/1.0% FCS (v/v)). After washing with PBS, the fluorescent intensity was measured with a FACSCalibur flow cytometer (BD Biosciences), and the data were analyzed using WINMDI software (Scripps, La Jolla, CA, USA). The results are expressed in terms of the relative mean fluorescence intensity (MFI) on CD11c^+^ gated conventional dendritic cells.

### 2.5. Cellular Morphology Analysis

The morphology of the DC cells was examined on day 6 and 24 h after LPS or WE-CN stimulation using a phase contrast microscope and digital photography to record the images.

### 2.6. Cytokine Detection

To quantify the production of cytokines and chemokines, supernatants were collected from immature DCs propagated in the presence of LPS or WE-CN. After incubation, cytokines (mTNF-alpha, mIL-6, mIL12p70, and mIL-4) production by DCs was measured in the supernatants by sandwich ELISA assays according to the manufacturer's specifications (all from PeproTech, Rocky Hill, NJ, USA). 

### 2.7. Allogenic Mixed Lymphocyte Reaction

Mouse T cells were isolated from naïve C57BL/6 mice spleens using EasySep Mouse T Cell Enrichment Kitseparation, respectively, according to the manufacturer's protocol (Stem Cell Technologies, Grenoble, France). Immature DCs were stimulated with LPS (100 ng/mL) or CN-WE (100 *μ*g/mL) for 24 hr, and then the cells were harvested, washed, and diluted with the prepared enriched T cells (2 × 10^5^) in ratios of 1 : 25, 1 : 5, and 1 : 1 (DC : T) in 96-well round-bottom plates (Corning). After an additional 96 hr of coincubation, cell proliferation was assessed by adding 1 *μ*Ci of [^3^H] thymidine (GE Healthcare, Buckinghamshire, UK) for overnight incubation; quantification of incorporated [^3^H] thymidine was subsequently performed by liquid scintillation counting on a *β*-Counter (Beckman Instruments, Palo Alto, CA, USA).

### 2.8. OVA-Specific T-Cell Activation

The protocol for OVA-specific T-cell activation was modified from our previous report [23, 24]. Briefly, immature DCs were pulsed with 2 *μ*g/mL OVA_257–264_ (OVAP_1_) or OVA_323–339_ (OVAP_2_) (synthesized by Echo Chemical Co., Taiwan) in the presence of LPS or WE-CN (100 *μ*g/mL) for 24 hr. CN was then washed out, and OVAP_1_-specific CD8^+^ T cells (2 × 10^5^) and OVAP_2_-specific CD4^+^ T cells (2 × 10^5^) were added to the culture at various DC : T cell ratios (as indicated) in 96-well round-bottom plates. The OVA-specific cells were positively enriched from the spleens of OT-1 and OT-2 mice by EasySep Mouse CD8a Positive Selection Kit separation and EasySep Mouse CD4 Positive Selection Kit separation, respectively, according to the manufacturer's protocol (Stem Cell Technologies, Grenoble, France). After an additional 72 hr of coincubation, T-cell proliferation was measured by adding 1 *μ*Ci of [^3^H] thymidine (GE Healthcare, Buckinghamshire, UK) for overnight incubation; quantification of incorporated [^3^H] thymidine was subsequently performed by liquid scintillation counting on a *β*-Counter (Beckman Instruments, Palo Alto, CA, USA). In addition, the IFN-*γ* cytokine levels in the supernatants from DC-OT-I/OT-II cultures were determined by a sandwich IFN-*γ* ELISA kit (eBioscience San Diego, CA, USA) according to the manufacturer's protocol.

### 2.9. Western Blot Analysis

Immature DCs were stimulated with 100 *μ*g/mL WE-CN, and whole cell lysates were prepared at the indicated time points as previously described [23]. The protease inhibitors leupeptin (Sigma-Aldrich, St. Louis, MO, USA) and aprotinin (Sigma-Aldrich, St. Louis, MO, USA) were used at a concentration of at 10 *μ*g/mL in all steps. Protein concentrations were determined using a BCA protein assay kit (Pierce Rockford, IL) prior to western blot or other protein analyses.

For protein detection, protein extracts (40 *μ*g/mL) were boiled separated on 10% SDS-polyacrylamide gels and electrotransferred to nitrocellulose membranes. The membranes were blocked for 1 hr with 5% skim milk in TBS + 0.05% Tween 20. After blocking, the membranes were incubated overnight with the suggested concentrations of either a primary antibody against phospho-p38 (no. 9211), p38 (no. 9212), phospho-p42/44 (no. 9101), total p42/44 (no. 9102), phospho-JNK (no. 4688), and b-Actin (no. 4970) (all purchased from Cell Signaling Technology) or total JNK (SC-571) (purchased from Santa Cruz Biotechnology, Danvers, MA, USA). The membranes were washed prior to incubation with HRP-Conjugated Goat Anti-Mouse IgG or HRP-Conjugated Goat Anti-Rabbit IgG secondary Abs (Jackson ImmunoResearch, West Grove, PA, USA). The proteins were detected by enhanced chemiluminescence (GE Healthcare, UK) and analyzed using the LAS3000 system (Fujifilm, Tokyo, Japan). Densitometric analysis was performed with ImageJ software (National Institute of Health, Bethesda, MD, USA). Active forms of pErk, p38, and pJNK were normalized with corresponding total nonphosphorylated forms.

### 2.10. Preparation of Nuclear Extracts and NF-*κ*B Activity Assay

Nuclear extracts were prepared using the NE-PER Nuclear and Cytoplasmic Extraction system (Pierce Rockford, IL, USA) according to the manufacturer's instructions. Protein concentrations were determined using a BCA protein assay kit (Pierce, Rockford, IL, USA). For each assay, 5 mg/mL of nuclear extracted was used in a TransAM NF-*κ*B p65 ELISA kit (Active Motif, Carlsbad, CA) according to the manufacturer's instructions.

### 2.11. Therapeutic Efficacy in a Mouse Model of Established MBT-2 Tumors

The MBT-2 tumor model has previously been described [22]. Briefly, MBT-2 cells (1 × 10^6^) in 0.5 mL of cold PBS were injected subcutaneously (s.c.) into the flanks of female C3H/HeN mice. Ten days after injection (when the tumors were palpable), the mice were injected intramuscularly in the left hind thigh muscles with 100 *μ*g of the pRC/CMV DNA plasmid (control group), 100 *μ*g of a HER-2/neu DNA vaccine (pRC/CMV vector carrying the cDNA encoding the extracellular domain of the human HER-2/neu) [25] in 100 *μ*L 0.9% NaCl, 100 *μ*g of the pRC/CMV DNA plasmid mixed with 100 *μ*g WE-CN, or 100 *μ*g of the HER-2/neu DNA plasmid mixed with 100 *μ*g WE-CN. The mice were injected three times at weekly intervals. The effects of these treatments on the growth of MBT-2 tumors were then monitored twice a week. Tumor volume was calculated using the formula for a rational ellipse: (*m*_1_ × *m*_2_ × *m*_2_ × 0.5236), where *m*_1_ represents the longer axis and *m*_2_ represents the shorter axis. The mice were euthanized when the tumor size reached >2500 mm^3^ in mean diameter or the mouse was in poor condition and death was imminent. The significance of differences in survival was tested by the Kaplan-Meier analysis (GraphPad Prism 4.0, La Jolla, CA. USA).

### 2.12. Quantitative RT-PCR

The quantitative RT-PCR protocol was modified from our previous report [24, 26]. Three days after the last of DNA vaccination, spleen cells (2 × 10^6^ cells/well) from mice from the different treatment groups were stimulated with recombinant HER-2/neu protein (10 *μ*g/mL, R&D Systems) in a 24-well plate for 48 hr. After stimulation, CD4^+^ T cells were purified by positive selection (purity >90%) (Stem Cell Technologies, Grenoble, France). Then, total RNA was extracted using the RNAspin Mini Kit (GE Healthcare, Buckinghamshire, UK). cDNA was generated from denatured total RNA using a Transcriptor First Strand cDNA Synthesis Kit (Roche) according to the manufacturer's instructions. The specific primers were used as previously described [24, 26] and as described below: IFN-*γ*, F: 5′-ACTGGCAAAAGGATGGTGAC-3′ and R: 5′-ACCTGTGGGTTGTTGACCTC-3′; IL-4, F: 5′-TCAACCCCCAGCTAGTTGTC-3′ and R: 5′-AAATATHCHAAGCACCTTTGG-3′; hypoxanthine guanine phosphoribosyl transferase 1 (HPRT), F: 5′-GTTGGATAAGGCCAGACTTTGTTG-3′ and R: 5′-GATTCAACTTGCGCCATCTTAGGC-3′. An Eco Real-Time PCR System (Illumina) and SYBR Green Master Mix (Roche) were used for quantitative real-time PCR. The gene expression was normalized to HPRT expression. These normalized values were then expressed relative to the control group.

### 2.13. Detection of Antigen-Specific CD8^+^/IFN-Gamma^+^ T Lymphocytes in Spleen Cells

The protocol for detection of antigen-specific CD8^+^/IFN-gamma^+^ T lymphocytes was modified from our previous report [23, 26]. After the last DNA vaccination for three days, RBC lysis buffer (eBioscience)-lysed, single-cell spleen suspensions (2 × 10^6^ cells/well) from mice from the different vaccination groups were pulsed for 18 hr with a peptide pool composed of 10 *μ*g/mL each of peptide 362–370 (EFAGKKI) (BioBasic, Canada) and peptide 404–412 (EEITGYLYI) (BioBasic, Canada) of the human HER-2/neu sequence. Brefeldin A (10 *μ*g/mL) (Sigma St. Louis, MO, USA) was added during the last 6 hr of culture. After stimulation, the cells were harvested and surfaced-stained with phycoerythrin- (PE-) conjugated CD8^+^ (CLONE 53-6.7) (eBioscience). The cells were subsequently fixed/permeabilized (Cytofix/Cytoperm Plus; BD Biosciences) and stained with FITC-conjugated anti-IFN-gamma (XMG1.2) (eBioscience). The percentage of CD8^+^IFN-gamma^+^ double positive cells among gated CD8+ T cells was measured using a FACSCalibur flow cytometer (BD Biosciences), and the data were analyzed using WINMDI software (Scripps, La Jolla, CA, USA). The CD8^+^ T-cell gating strategy was as follows: the lymphocyte population of the spleen cells was gated on low forward scatter (FSC) versus side scatter (SSC) dot plots, and the FITC-labeled CD8^+^ cells were further gated within the lymphocyte gate. Cells that fulfilled these criteria were acquired (1 × 10^4^).

### 2.14. Statistical Analysis

All statistical analyses were performed using the GraphPad Prism software package version 4.0. The statistical analyses of cytokine production, surface marker expression, T-cell proliferation, tumor size, and percentage of CD8^+^/IFN-gamma^+^ T lymphocytes, western blotting, were performed using one-way ANOVA followed by Tukey's post hoc test. For analysis of the mouse survival rates, the Kaplan-Meier method was used, and statistical significance was determined by log-rank analysis. Values of *P* < 0.05 were considered to be statistically significant. 

## 3. Result

### 3.1. WE-CN Can Induce BMDCs Phenotypic Maturation

**Figure 1:**
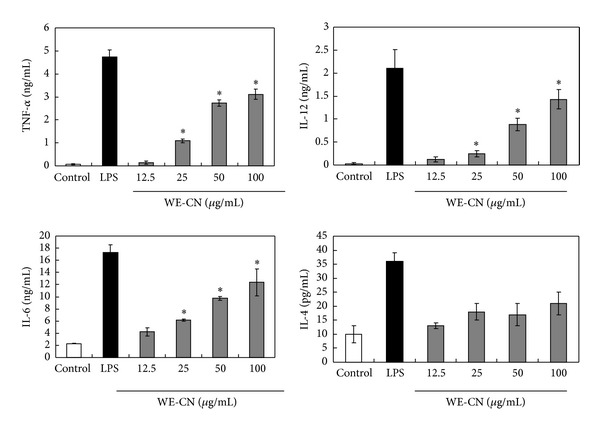
Cytokine production is increased in BMDCs stimulated with WE-CN. Immature BMDCs were stimulated with 100 ng/mL LPS or various concentrations of WE-CN. The control group was treated with PBS alone. Culture supernatants were collected after 24 hr (or 6 hr for TNF-alpha), and the levels of cytokine secretion were analyzed by ELISA. The data are presented as the means ± SD of samples from three wells. *P < 0.05 compared to control. All data are representative of three independent experiments showing similar results.

**Figure 2:**
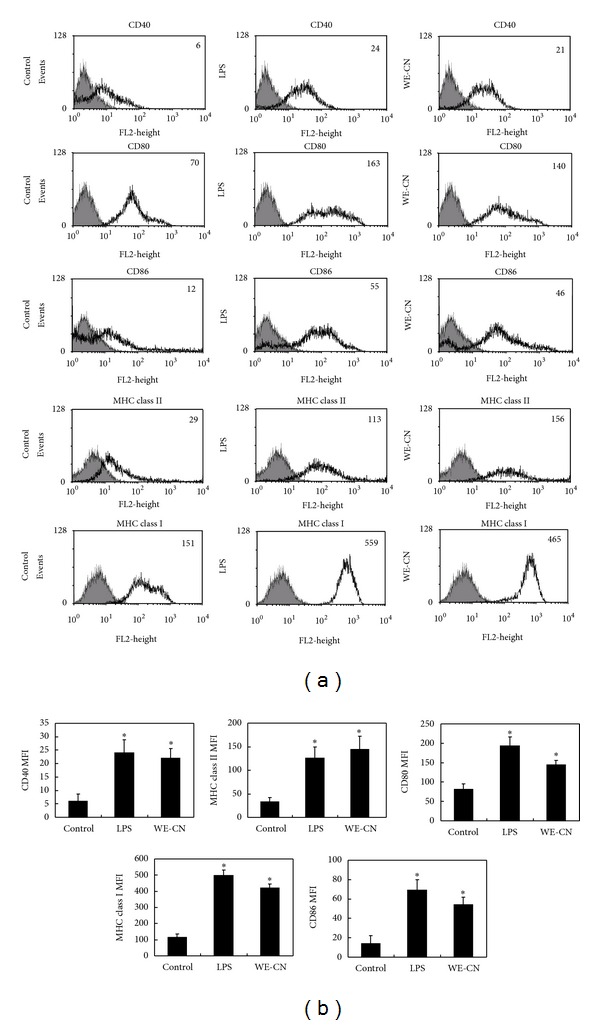
WE-CN upregulates the expression of immunomodulatory cell surface markers on BMDCs. Immature BMDCs were stimulated with 100 ng/mL LPS or 100 *μ*g/mL WE-CN for 24 hr. The control group was treated with PBS alone. After incubation, the expression of the surface markers CD40, CD80, CD86, MHC class I, and MHC class II was analyzed by flow cytometry with fluorescently labeled Abs. All data were gated on CD11c^+^ cells. The gray-filled area represents staining with an isotype-matched control Ab. (a) The histogram shows data from one representative experiment of each group. (b) The bar graphs represent the mean ± SD from three independent experiments.

**Figure 3:**
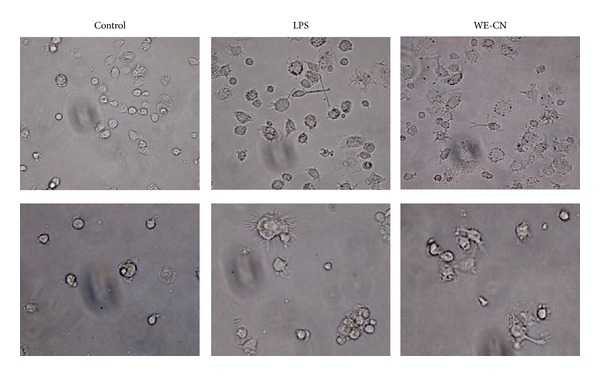
Morphology of the BMDCs treatment with PBS, LPS, or WE-CN under a light microscopy (×200).

Mature DCs are characterized by the synthesis and secretion of proinflammatory cytokines, and upregulation of surface costimulatory molecules and major histocompatibility complex molecules with important modulatory functions in inflammatory responses and adaptive immunity [1–3]. Therefore, in the first series of experiments, we investigated the effect of water extract of *Clitocybe nuda* (WE-CN) on the secretion of the selective proinflammatory cytokines TNF-alpha and IL-6, the Th1 cytokine IL-12, and the Th2 cytokine IL-4 in the supernatants of BMDCs by sandwich ELISAs. BMDCs treated with LPS were used as a positive control. As shown in Figure 1, incubation of DCs with WE-CN greatly increased the production of TNF-alpha, IL-6, and IL-12 in a dose-dependent manner, suggesting that WE-CN enhances the maturation and immunostimulatory activity of DCs. The maturation status of BMDCs was also indicated by the enhanced expression of surface molecules on CD11c+ cells. As shown in Figure 2, WE-CN (100 *μ*g/mL) stimulation of BMDCs resulted in significant upregulation of costimulatory molecules (CD80, CD86, and CD40) and major histocompatibility complex molecules (MHC class II and MHC class I) within 24 hr compared to untreated immature BMDCs. Therefore, morphological changes take place as well during the life cycle of DCs: immature DC precursors are often small, round-shaped cells that turn into larger cells with irregular shape and many cytoplasmic protrusions (dendrites) as the cell matures [27]. Thus, we also investigated the effect of WE-CN on the morphological change by phase contrast microscope. As shown in Figure 3, the shapes of LPS- and WE-CN-treated DCs were irregular and have more dendrites compared to the PBS-treated cells after 24-hour treatment. Taken together, these results confirming that WE-CN can induce DC phenotypic maturation. 

### 3.2. WE-CN Increases the Ability of BMDCs to Stimulate OVA-Specific T-Cell Proliferation

**Figure 4:**
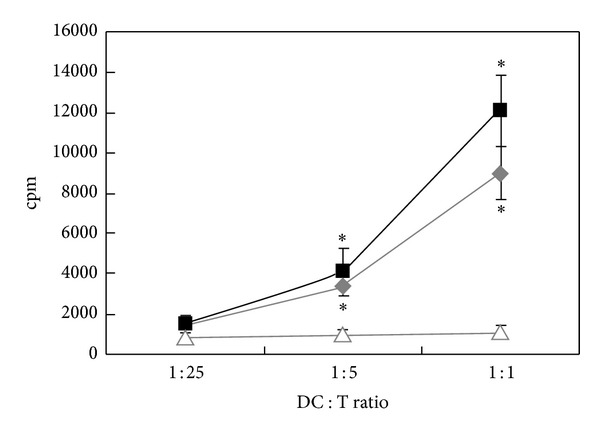
WE-CN induces the capability of stimulating allogeneic T-cell response in MLR of BMDCs. T cells were prepared from the spleens of naïve C57BL/6 mice. Purified T cells were then cocultured with PBS-, LPS- (100 ng/mL), or WE-CN- (100 *μ*g/mL) treated BMDCs at the indicated ratio of DC : T cells for 96 hr. Cell proliferation was measured by [^3^H]-thymidine incorporation for 18 hr. The data shown represent the mean ± SD of samples from three wells. *P < 0.05 compared to control. All data are representative of three independent experiments showing similar results.

**Figure 5:**
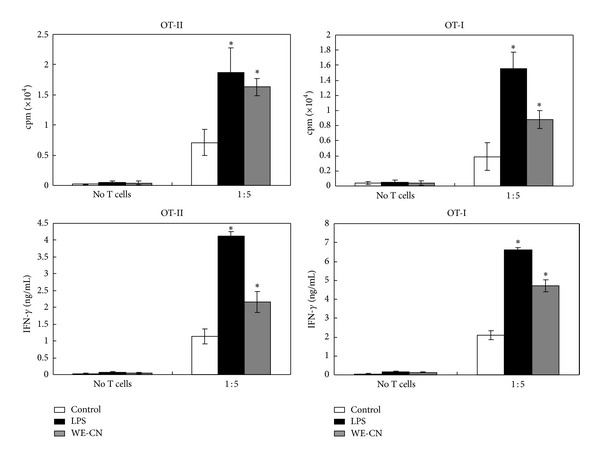
WE-CN-treated BMDCs increase T-cell activation in response to the specific antigen OVA. (a) CD8^+^ T cells and CD4^+^ T cells were prepared from the spleens of OT-I and OT-II mice, respectively. Purified T cells were cocultured with PBS-, LPS- (100 ng/mL), or WE-CN- (100 *μ*g/mL) treated DCs pulsed with an OVA peptide at the indicated ratio of DC : T cells for 96 hr. Cell proliferation was quantified by [^3^H]-thymidine incorporation for 18 hr. (b) Supernatants were collected from cultures after 4 days, and IFN-*γ* production was measured by ELISA. The data shown represent the mean ± SD of samples from three wells. *P < 0.05 compared to control. All data are representative of three independent experiments showing similar results.

A critical function of maturate DCs is to promote antigen-specific T-cell proliferation. Therefore, we examined the effect of WE-CN on BMDC-mediated allogeneic T-cell proliferation in a mixed lymphocyte reaction (MLR). As shown in Figure 4, at DC-to-T cells ratios of 1 : 1 and 1 : 5, WE-CN-treated DCs significantly induced more proliferation of naïve allogeneic T cells than those PBS-treated DCs did. To further compare the stimulatory properties of DC cultured with WE-CN, we next examined the effect of WE-CN on BMDC-mediated activation of OVA antigen-specific T-cell responses. CN-treated, OVA_257–264_ (OVAP_1_), or OVA_323–339_ (OVAP_2_) peptide-loaded BMDCs were cocultured with their allogeneic OVA-specific CD4^+^ OT-II or CD8^+^ OT-I T cells, and T-cell proliferation was measured by [^3^H] thymidine incorporation. Our results showed that WE-CN-activated BMDCs effectively induced enhanced OVA-specific CD4^+^ (OT-II) and CD8^+^ (OT-I) T proliferative responses in vitro (Figure 5). In addition, because IFN-*γ* is produced by activated T cells, the IFN-*γ* levels in the culture supernatants were also measured using ELISA. As shown in Figure 5, WE-CN treatment also increased the amount of INF-*γ* produced by the activated CD4^+^ and CD8^+^ T cells. These results revealed that WE-CN enhances the ability of DCs to induce Ag-specific T-cell immune responses. 

### 3.3. WE-CN Increases MAPK and NF-*κ*B Pathways Activation in BMDCs

**Figure 6:**
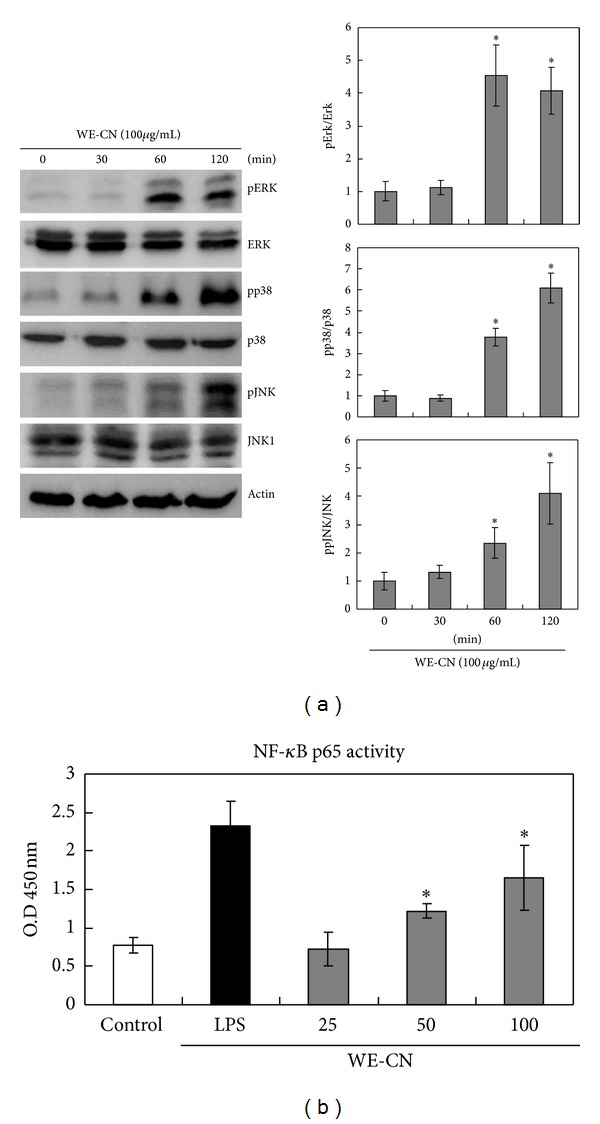
WE-CN induces MAPK phosphorylation and NF-*κ*B binding activity in BMDCs. Immature BMDCs were treated with WE-CN (100 *μ*g/mL), and whole cell lysates or the cytosolic fraction of lysates was collected at the indicated time points. (a) MAPK phosphorylation levels were analyzed by western blot analysis with anti-ERK, anti-JNK, and anti-p38 MAPK antibodies (against both phosphorylated and nonphosphorylated forms of the proteins). pErk, pp38, and pJNK 2 levels were normalized with total Erk, p38, and JNK levels and then compared with the control group arranged as one unit. The data represent the mean ± SD of samples from three independent experiments. (b) The NF-**κ**B assay was described in Section 2. The NF-**κ**B binding activity is shown as relative OD450 levels. The data represent the mean ± SD of samples from three wells. *P < 0.05 compared to control. The data are representative of three independent experiments showing similar results.

As previously shown, the activation of MAPKs and NF-*κ*B plays a crucial role in DC maturation and response to inflammatory stimuli [28, 29]. To gain additional insight into the molecular mechanism underlying the effects of WE-CN, we sought to study the effect of WE-CN treatment on the MAPK and NF-*κ*B pathways in BMDCs. BMDCs were stimulated with WE-CN, and the levels of MAPK phosphorylation were assessed by western blotting. Our results showed that WE-CN can upregulate the phosphorylation of the MAPKs ERK, p38, and JNK between 60 and 120 min after treatment, whereas the levels of the nonphosphorylated proteins were not affected (Figure 6(a)). To further determine whether WE-CN increases the activation of NF-*κ*B, nuclear extracts were collected and analyzed for NF-**κ**B binding activity using an ELISA-based assay. As shown in Figure 6(b), CN treatment resulted in an increase in the nuclear translocation of p65 and upregulated NF-*κ*B binding activity at 3 hr after stimulation. Therefore, these results suggest that CN induces DC activation, possibly through activation of the MAPK and NF-*κ*B pathways, which partially explains the activating effect of WE-CN on DC maturation.

### 3.4. BMDCs from TLR-4/2-Deficient Mice Do Not Respond to WE-CN Stimulation

**Figure 7:**
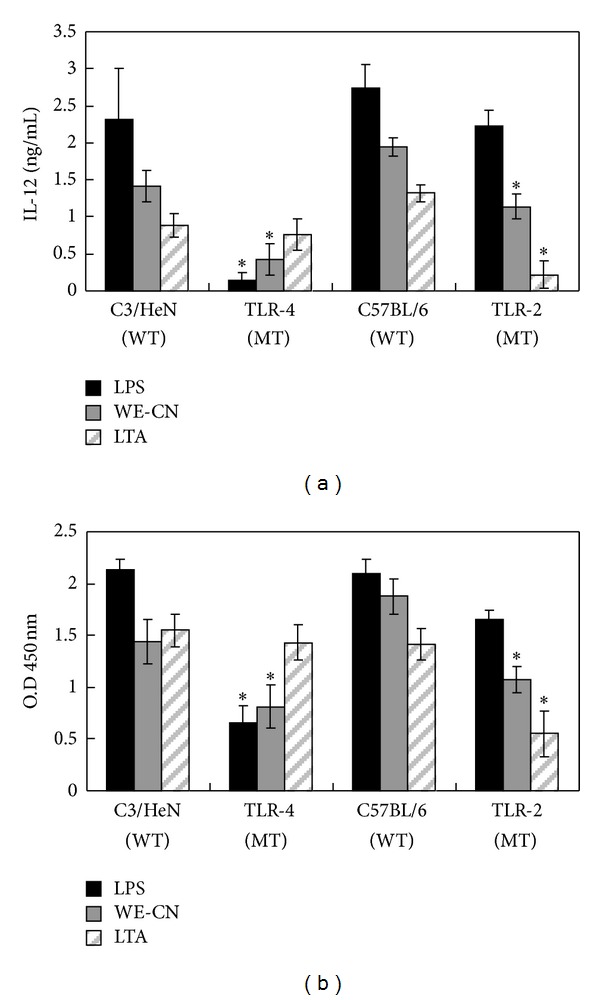
WE-CN induces IL-12 expression and NF-*κ*B activity through a TLR-4- and/or TLR-2-dependent signaling pathway. BMDCs were harvested from C3H/HeN, C3H/HeJ (TLR-4-deficient), C57BL/6, or TLR-2 KO mice and stimulated with WE-CN (100 *μ*g/mL), LPS (100 ng/mL) or LTA (1 *μ*g/mL). (a) IL-12 cytokine production and (b) NF-*κ*B binding activity were measured 24 hr later (or 3 hr later for the NF-*κ*B binding assay). The data shown represent the mean ± SD of samples from three wells. *P < 0.05 for the comparison between stimulated BMDCs from the mutant mice and their relevant wild-type control group. All data are representative of three independent experiments showing similar results.

The recognition of certain mushroom compounds, such as water soluble polysaccharide proteoglycans and proteins, by TLR-2 and TLR-4 has been demonstrated in DCs and is required for optimal DC activation [24, 25, 30, 31]. Therefore, we further assessed whether TLR-2 and/or TLR-4 receptors were involved in WE-CN-induced DC activation. The TLR-4-deficient (C3H/HeJ) and TLR-2^−/−^ (C57BL/6 background) mouse strains are nonresponsive to LPS and lipoteichoic acid (LTA) due to a point mutation or knockout of their TLR-4 or TLR-2 receptor genes, respectively [25, 30, 31]. We used these mice to further characterize the function of TLR-4 or TLR-2 in WE-CN-mediated DC stimulation. Bone marrow-derived DCs from C3H/HeJ or TLR-2^−/−^ mice and wild-type C3/HeN or C57BL/6 mice were prepared and phenotypically analyzed. As shown in Figure 7(a), LPS and LTA did not induce significant IL-12 production in TLR-4-deficient or TLR-2^−/−^ BMDCs, respectively. In addition, WE-CN stimulated IL-12 production in a dose-dependent manner in wild-type DCs as expected; however, IL-12 production was dramatically reduced in BMDCs from both TLR-4-deficient and TLR-2 KO mice. We next examined whether the nonresponsiveness of DCs from TLR-4 mutant or TLR-2 KO mice to WE-CN-stimulation is functionally related to their NF-*κ*B activity. As shown in Figure 7(b), the NF-*κ*B activity of the DCs from TLR-4 and TLR-2 mutant mice was significantly lower upon WE-CN treatment compared with wild-type DCs. Taken together, these results show that WE-CN can induce BMDC maturation through interaction with TLR-4 and/or TLR-2 molecules.

### 3.5. WE-CN Enhanced the Antitumor Effect of a HER-2/neu DNA Vaccine

**Figure 8:**
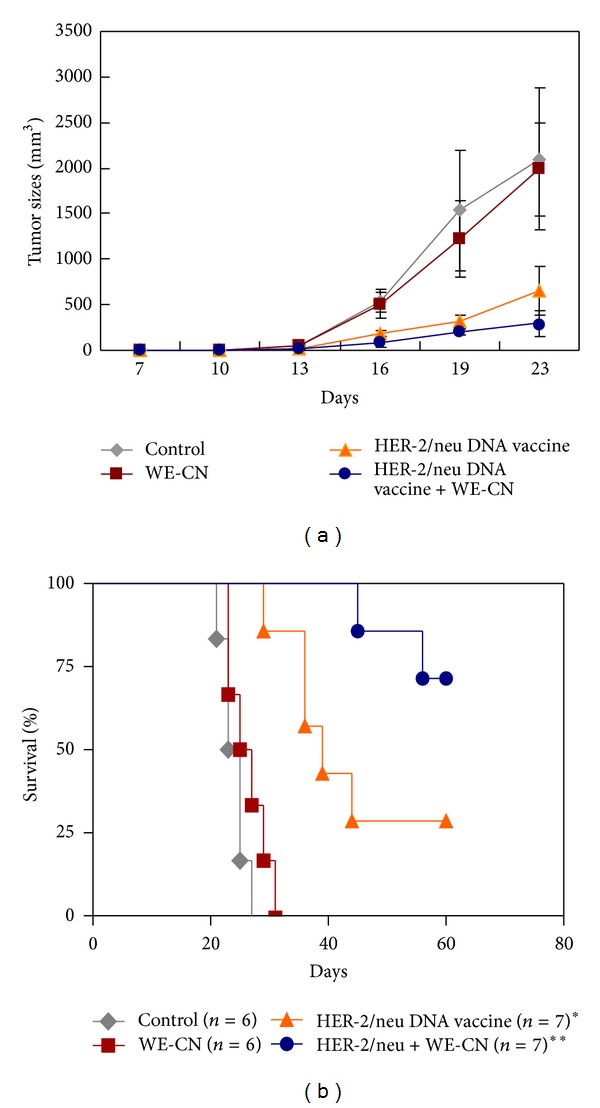
WE-CN enhances the therapeutic effects of a HER-2/neu DNA vaccine against MBT-2 tumors in C3H/HeN mice. Ten days after s.c. MBT-2 inoculation, the tumor-bearing mice were vaccinated with the indicated formulas. (a) The tumor volumes were measured at the indicated times. The mean values ±SD from six to seven mice per group are shown. (b) The Kaplan-Meier survival curve for the different groups of mice is shown. The symbol * indicates a statistically significant difference compared with control group (*P* < 0.05). The symbol ** indicates comparison with the HER-2/neu DNA vaccine alone group (*P* < 0.05). The data are representative of three independent experiments with similar results.

We have previously demonstrated that an intramuscular HER-2/neu DNA vaccine has a therapeutic effect on established p185^neu^-expressing MBT-2 tumors in C3H/HeN mice [22, 32, 33]. Using this model, we further examined whether WE-CN can increase the efficacy of a HER-2/neu DNA vaccine. As shown in Figure 8(a), immunization with either the control vector alone or control vector plus 100 *μ*g/mL WE-CN did not exert therapeutic effects on MBT-2 tumor growth, and all mice in these two groups had to be sacrificed due to the size of their tumors or their overall health; these mice were expected to have become moribund by day 35 at the latest. In contrast, when the mice were vaccinated with the HER-2/neu DNA alone or HER-2/neu DNA plus WE-CN (100 *μ*g), the growth of the tumors was significantly reduced compared to tumors at earlier time points. However, injection with HER-2/neu DNA plus WE-CN markedly prolonged survival compared to the HER-2/neu DNA alone (Figure 8(b)). These results clearly indicate the additional benefit of CN on the therapeutic efficacy of the HER-2/neu DNA vaccine.

### 3.6. WE-CN Promoted the Th1 Immune Responses Induced by HER-2/neu DNA Vaccination

**Figure 9:**
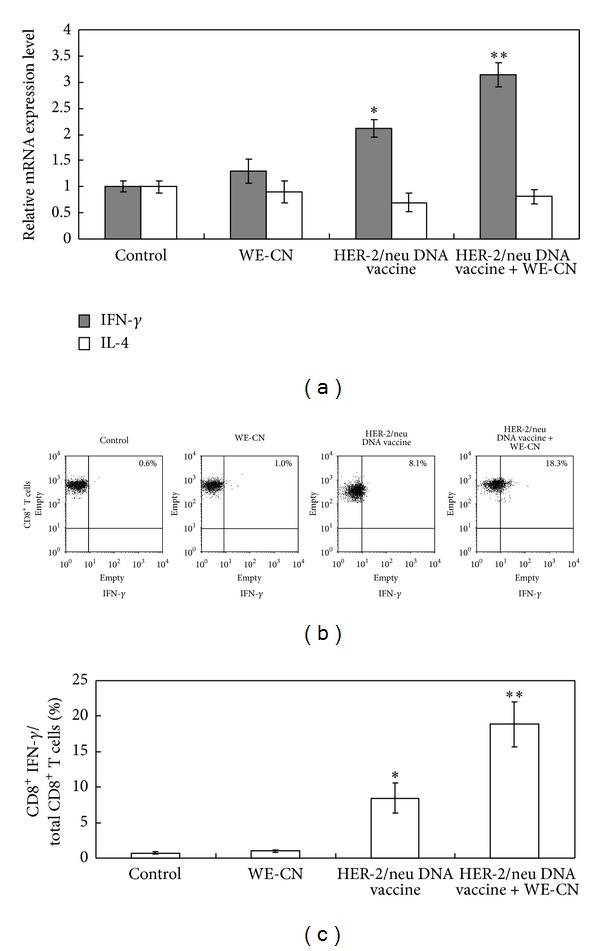
WE-CN promotes CD4^+^Th1 differentiation and increased HER-2/neu-specific functional CD8^+^ T cells in spleens from vaccinated mice. (a) CD4^+^ T cells were purified from the different groups and stimulated with recombinant HER-2/neu protein (10 *μ*g/mL). After 3 days, the expression levels of IFN-*γ* or IL-4 mRNA were determined by quantitative real-time RT-PCR. The data were normalized to HPRT expression in each sample, and error bars indicate the mean ± SD of six mice per group assessed from three independent experiments. (b) The spleen cells from the different vaccinated groups were stimulated with a peptide pool composed of 10 *μ*g/mL each of peptide 362–370 (EFAGKKI) (BioBasic, Canada) and peptide 404–412 (EEITGYLYI) of the HER-2/neu sequence. After stimulation for 18 hr, the percentage of IFN-gamma-producing CD8+ T cells was determined by flow cytometry. The dot plot shows data from one representative mouse from each group. The bar graph represents the mean ± SD of five mice from two independent experiments. The symbol * indicates a statistically significant difference compared to control vector-treated mice (*P* < 0.05). The symbol ** indicates a statistically significant difference compared to the HER-2/neu DNA vaccine alone group (*P* < 0.05).

Our previous study demonstrated that Th1-based immune responses, specifically the generation of cytotoxic CD8^+^ T cells, represent the primary immunological mechanism for the suppression of the implanted MBT-2 tumors in mice [22–24, 26, 32, 33]. In addition, we have shown that WE-CN-stimulated DCs preferentially release the Th1-dominant cytokine IL-12 (Figure 1) and facilitate Th1 CD4^+^ differentiation (Figure 5). Therefore, we hypothesized that WE-CN promotes Th1 immune responses that consequently enhance the antitumor immunity resulting from HER-2/neu DNA vaccination. To test this possibility, spleen cells collected from mice in the different vaccination groups were stimulated with recombinant HER-2/neu protein, and the expression of IFN-*γ* (Th1) and IL-4 (Th2) within purified CD4^+^ T cells was determined by a qPCR assay. As shown in Figure 9(a), mice immunized with the HER-2/neu DNA vaccine-CN combination showed significantly higher levels of IFN-*γ* than those immunized with the HER-2/neu DNA vaccine alone; no significant differences were observed in IL-4 production. To further examine the HER-2/neu-specific CD8^+^ responses in vaccinated mice, we used flow cytometry to analyze the intracellular expression of IFN-*γ* in CD8^+^ T cells. As shown in Figure 9(b), spleen cells collected from mice immunized with the HER-2/neu DNA vaccine/WE-CN combination generated more HER-2/neu-specific CD8^+^ IFN-gamma T cells than those of the mice immunized with the HER-2/neu DNA vaccine alone. These results suggest that coadministration of the HER-2/neu vaccine with WE-CN may elicit stronger HER-2/neu-specific Th1 responses induced by HER-2/neu DNA vaccination, which could enhance the antitumor efficacy of the HER-2/neu DNA vaccine. 

## 4. Discussion 

Dendritic cells (DCs) are a crucial cell type that acts at the interface of innate and adaptive immune responses and has the unique ability to activate naive T cells [1–3]. Potent modulation of the activation and function of this essential cell type might have potential efficacy against tumor or virus infection or represent a candidate adjuvant approach for application in immunotherapy and vaccination [4]. In this study, we examined the activity of water extract of *C. nuda* (WE-CN) on the immune function of DCs. Our results show that WE-CN induces DC maturation, based on the levels of costimulatory molecules and cytokines, and promotes the activation of allogeneic T cells as the primary DC function. This study is the first to report that *C. nuda* has immunostimulatory activity on immune systems.

DCs are believed to regulate T-cell-mediated immunity. In addition, the type of cytokines synthesized and released by DCs upon activation is believed to play an important role in determining the fate choice of naïve CD4^+^ T cells, which can be subdivided into Th1, Th2, and Th17 cells and Treg cells [34]. Specifically, IL-12 is a pivotal cytokine for Th1 differentiation, whereas IL-4 inhibits Th1 but promotes Th2-type responses [35]. In the present study, we observed that WE-CN can significantly increase IL-12 production but does not affect IL-4 production in BMDCs (Figure 1). In addition, we also showed that coculture of WE-CN-stimulated OVA peptide-pulsed BMDCs and OVA-specific T cells can significantly increase IFN-*γ* production (Figure 5), which is a major product of Th1 cells [36]. Thus, these results indicate that WE-CN-stimulated DCs preferentially promote Th1 immune responses. Therefore,* C. nuda* may be effective in antitumor or antiviral effects or for the treatment of allergic diseases (which are Th2-dominant immune responses) by resultant augmentation of Th1 cells.

Recently, IL-17-producing (Th17) cells have been identified as a novel class of helper CD4^+^ T cells [37]. IL-17 plays an important role in host defense against bacterial or fungal infections [38–41]. In addition, Th17 is also related to the pathogenesis of many chronic inflammatory diseases and autoimmune diseases [42, 43]. It has been shown that transforming growth factor-*β* (TGF-*β*), IL-6, IL-21, and IL-23 are involved in Th17 differentiation [44, 45]. We found that WE-CN significantly enhances the IL-6 production by DCs, suggesting that Th17 may be induced by WE-CN. However, we focused on the adjuvant effect of WE-CN and did not examine the induction of Th17 in this study. 

Our present study showed that active MAPKs (ERK, p38, and JNK) and NF-*κ*B signaling pathways result in DC maturation (Figure 6). Because NF-*κ*B binding sites are found in the promoter regions of various proinflammatory cytokines, including TNF-alpha, IL-6, and IL-12 [46–48], and of costimulatory molecules, such as CD40 and CD86 [49, 50], the NF-*κ*B pathway may be involved in WE-CN-induced DC activation. However, the reported role of the MAPK signaling pathway in DC maturation is controversial. p38 mitogen-activated protein kinase is well known to play an important role in DC maturation [29]. Lin et al. have shown that the ERK signaling pathway is required for DC maturation and cytokine production by polysaccharide purified from *Ganoderma lucidum* [51]. In contrast, the ERK pathway has also been shown to lead to differentiation of tolerogenic DCs [52]. Moreover, a previous study has shown that ERK and JNK signaling pathways activation in DCs favors a Th2 response, whereas p38 pathway activation favors a Th1 response [53–55]. One possible explanation is that the duration and intensity of MAPK signaling activation during DC maturation may differ in response to different stimuli, and different activation patterns of multiple signaling pathways may result in varied consequences. Further studies using pharmacological inhibitors of the MAPK pathway or overexpression of small interference RNA and dominant-negative gene strategies are required to clarify the importance of the intensity and duration of p38, ERK, and JNK phosphorylation in WE-CN-induced DC activation.

Recent data have suggested that stimulation through toll-like receptors (TLRs) is required for the innate immune response and optimal DC activation [56]. In the present study, using BMDCs from TLR-4 and TLR-2 mutant mice, we found that WE-CN induced DC maturation at least in part through interaction with TLR-4 and TLR-2 (Figure 7). The specific components of WE-CN that contribute to the observed effect on DCs remain unclear. Previous studies have shown that water-soluble polysaccharides, specifically *β*-1,3 glucans with *β*-1,6 linked side chains, proteoglycans, and proteins (such as fungal immunomodulatory protein (FIP) isolated from a various types of edible mushrooms), display similar effects on DCs by acting on toll-like receptor-2 and/or -4 [24, 25, 30, 31, 57, 58]. Therefore, we suggest that the effects of WE-CN on DC activation may be attributed to the polysaccharide, proteoglycan, and/or protein content of WE-CN; however, all of these possibilities require further study. 

We identified CN water extracts as novel ligands for TLR-4 and TLR-2. Because TLR-4 is one of the major components of the receptor for LPS and concentrations as low as 50 pg/mL are sufficient to promote DC activation [59], the possibility of contamination with the endotoxin LPS in the CN preparations used in this study required examination. LAL assays showed endotoxin content of <0.1 ng/mL in our 1 mg/mL CN stock solutions, resulting in a maximum possible contamination throughout the assays of 0.01 ng/mL in the 100 *μ*g/mL concentration of WE-CN. Titration experiments with purified LPS (*Escherichia coli*, serotype O26:B6) were performed and indicated that DCs under the culture conditions used in our experiments failed to mature in response to LPS concentrations of <0.1 ng/mL. (see supplemental Figures  1(a) and  1(b) in supplementary material available online at http://dx.doi.org/10.1155/2013/761454). Furthermore, in blocking experiments for LPS, BMDCs were incubated with 5 *μ*g/mL of the LPS inhibitor polymyxin B prior to WE-CN stimulation. Polymyxin B did not significantly affect the CN-induced IL-12 production and upregulation of CD80 but almost completely inhibited the LPS effect in the same experiment (supplemental Figure  2). Therefore, these data suggest that CN water extract causes DC maturation in part by binding to TLR-4 and/or TLR-2.

Our results showed that WE-CN can activate DCs in part by interaction with TLR-4/TLR-2 receptors (Figure 7). However, in addition to DCs, various other types of immune cells (including macrophages and B lymphocytes) can also express TLR and respond to TLR ligands [60]. Therefore, further examination of the direct effects of WE-CN on the functions of other immune cells, such as B cells and macrophages, is warranted.

DNA vaccines have been reported to generate high antigen-specific immunity in animal models for treating various diseases, including established tumors; however, poor immunogenicity in large animals and humans is the major obstacle for the practical use of these vaccines [61–63]. Therefore, the development of novel approaches to increase the immunogenicity of DNA vaccines and circumvent this limitation is urgently needed. To this end, combining DNA vaccination with strong adjuvants is one strategy to enhance the immunogenicity of DNA vaccines [64]. Our present study showed for the first time that the addition of WE-CN (100 *μ*g) significantly enhanced the antitumor effect of a HER-2/neu DNA vaccine on tumor growth, resulting in a higher survival rate compared to the HER-2 DNA vaccine alone group (Figure 8). Further studies revealed the mechanisms by which WE-CN induces antigen-specific Th1 responses, including increased functional CD8^+^ T cell populations in spleen cells and induction of elevated IFN-gamma mRNA expression levels compared to the HER-2 DNA vaccine alone group (Figure 9). Therefore, these data (corresponding to the results in Figures 1 and 5) suggest that WE-CN-stimulated DCs preferentially promote Th1 immune responses. Moreover, although we used HER-2/neu as a model antigen to demonstrate the adjuvant effects of WE-CN in this study, we propose that our results are encouraging for the application of *C. nuda* extract to enhance the potency of DNA vaccines or other immunotherapies for the control of other cancers and infectious diseases.

In summary, for the first time, we present evidence demonstrating that water extract of *C. nuda* can augment DC maturation in an in vitro culture system and contains adjuvant activity for DNA vaccines and therapeutic antitumor potential in a tumor-bearing mouse model. We suggest that these data may highlight the nutritional and medical value of *C. nuda* and can be used as the basis for further research. However, additional issues still warrant future investigation. For example, in this study, because we used a crude water extract of whole *C. nuda* concentrate, the identity of the specific components of the *C. nuda* extract that contribute to the observed effect on DCs is unclear. Therefore, further studies are required to identify the specific active component(s) that is responsible for the observed effect of WE-CN. In addition, we employed an in vitro culture model in this study to determine the effect of WE-CM on BMDC maturation, but we are aware that an in vivo (oral administration and feeding) design may be more applicable for defining the effects of dietary mushrooms as a nutritional intervention. Thus, further examination of whether the in vitro effects can be recapitulated in vivo (through oral administration and feeding) will be very important.

## Supplementary Material

Supplemental Figure 1: Titration experiments with purified LPS on BMDCs activation:
The result showed that DCs under the culture conditions used in our experiments failed to mature in response to LPS concentrations of <0.1 ng/mL.
Supplemental Figure 2. The stimulatory activity of WE-CN on BMDCs is not due to contamination:
Polymyxin B (5 *µ*g/ml) did not significantly affect the CN–induced IL-12 production and upregulation of CD80 but almost completely inhibited the LPS effect in the same experiment.
