# Supplementary figures and images for: Clitocybe nuda Activates Dendritic Cells and Acts as a DNA Vaccine Adjuvant
Source: Evid Based Complement Alternat Med. 2013 Aug 22;2013:761454. doi: 10.1155/2013/761454 (PMC3766593; doi:10.1155/2013/761454)

**Supplemental figure 1**

**A**

**
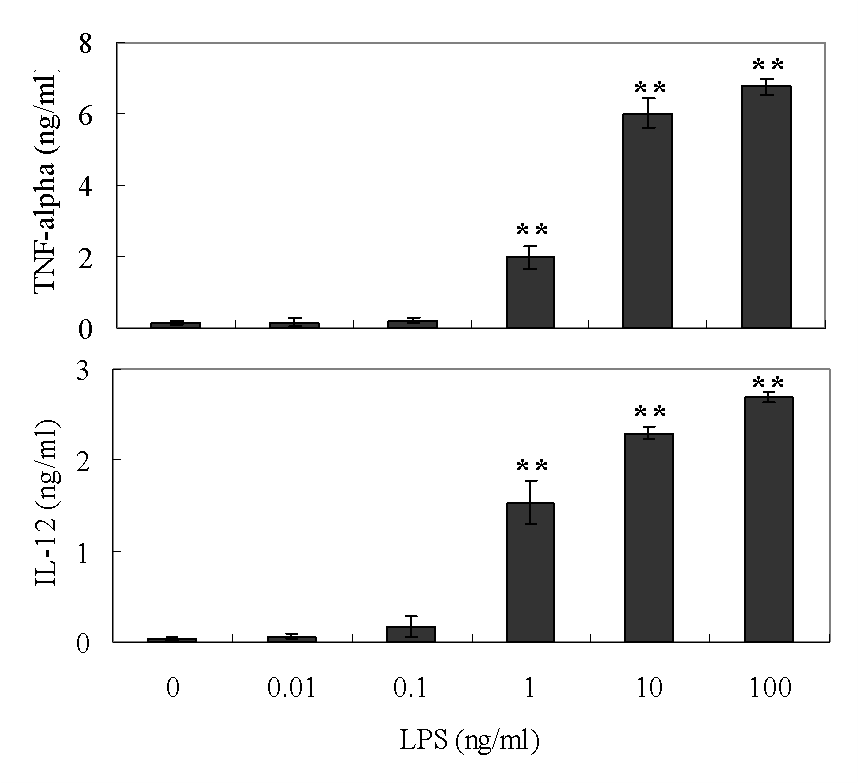
**

**B**

**
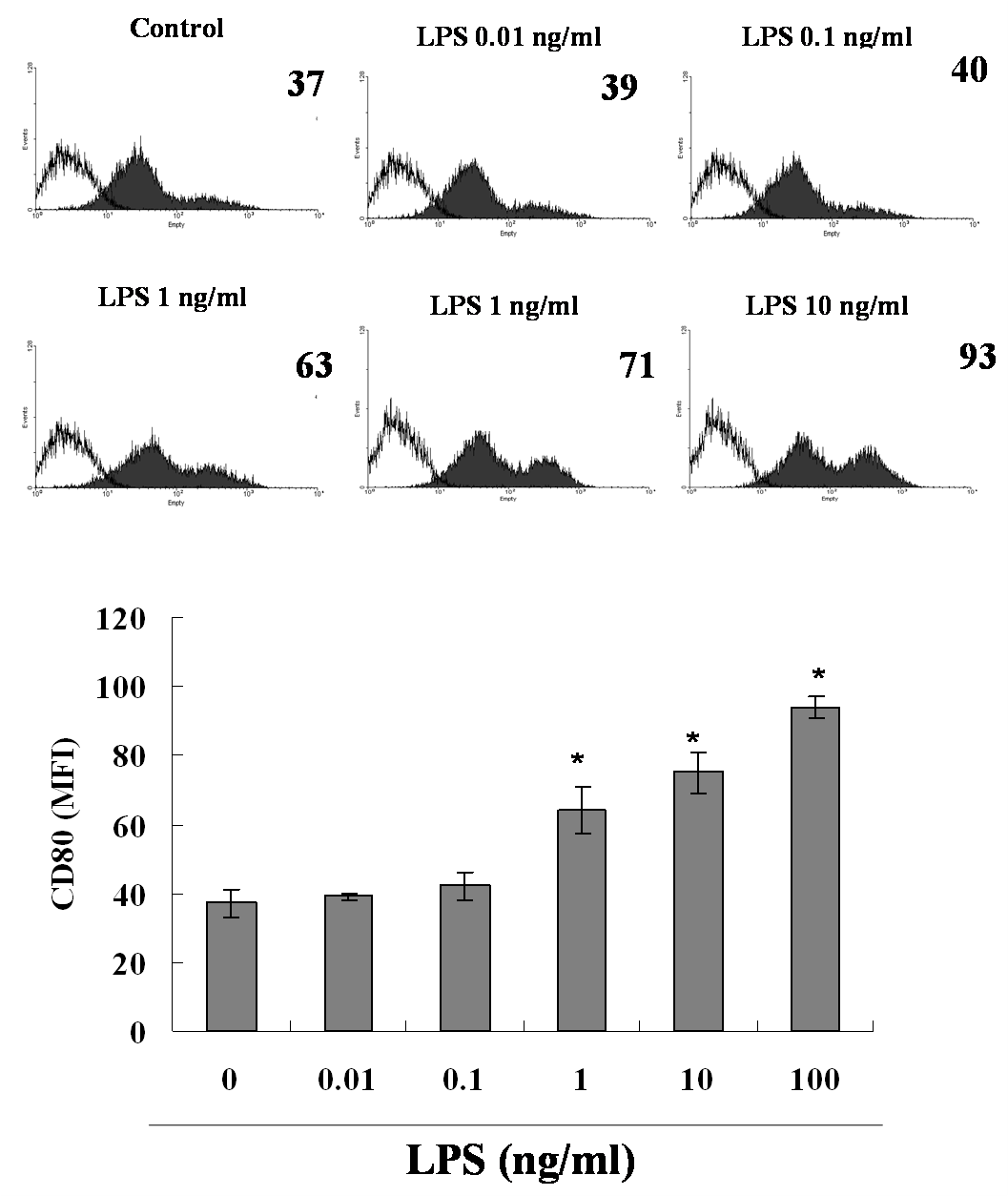
**

Supplement: Supplementary file 1 — Supplemental Figure 1: Titration experiments with purified LPS on BMDCs activation: The result showed that DCs under the culture conditions used in our experiments failed to mature in response to LPS concentrations of <0.1 ng/mL. Supplemental Figure 2. The stimulatory activity of WE-CN on BMDCs is not due to contamination: Polymyxin B (5 µg/ml) did not significantly affect the CN–induced IL-12 production and upregulation of CD80 but almost completely inhibited the LPS effect in the same experiment. [file 761454.f1.doc]

**Supplemental figure 2**

**A**


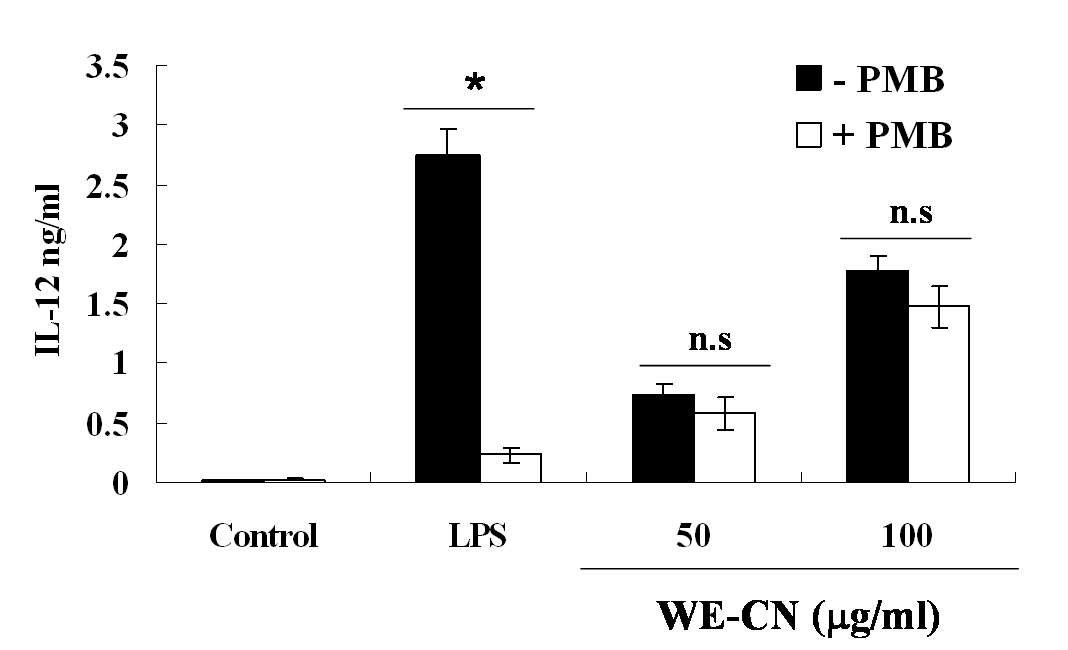


**B**

**
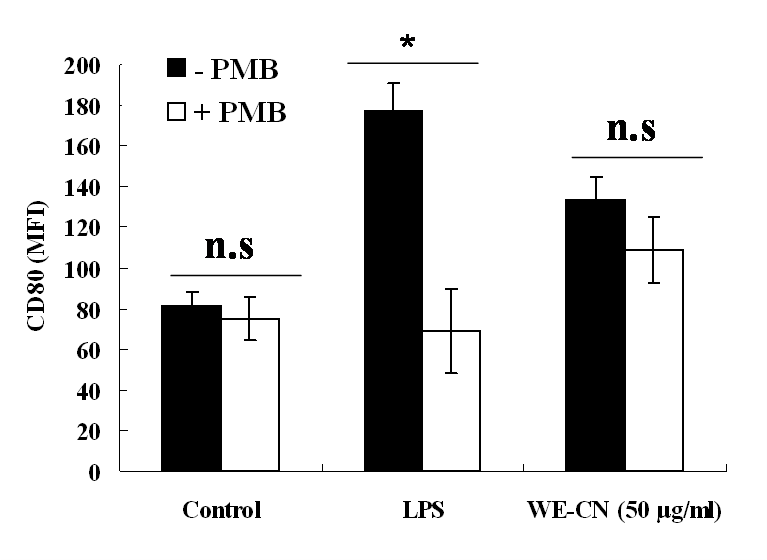
**

Supplement: Supplementary file 2 [file 761454.f2.docx]
